# The Chinese version of the Pediatric Quality of Life Inventory™ (PedsQL™) 3.0 Asthma Module: reliability and validity

**DOI:** 10.1186/1477-7525-9-64

**Published:** 2011-08-07

**Authors:** Lifen Feng, Yingfen Zhang, Ruoqing Chen, Yuantao Hao

**Affiliations:** 1 Department of Medical Statistics and Epidemiology, Center for Health Information Research, School of Public Health, Sun Yat-sen University, Guangzhou 510080, the People's Republic of China; 2 Special Center, the First Affiliated Hospital of Sun Yat-sen University, Guangzhou 510080, the People's Republic of China; 3 Department of Epidemiology, Key Lab of Public Health Safety of the Ministry of Education, School of Public Health, Fudan University, Shanghai 200032, the People's Republic of China

**Keywords:** Asthma, Children, Health-related quality of life, Reliability, Validity, PedsQL

## Abstract

**Background:**

Health-related quality of life (HRQOL) has been recognized as an important health outcome measurement for pediatric patients. One of the most promising instruments in measuring pediatric HRQOL emerged in recent years is the Pediatric Quality of Life Inventory (PedsQL™). The PedsQL™ 3.0 Asthma Module, one of the PedsQL™disease-specific scales, was designed to measure HRQOL dimensions specifically tailored for pediatric asthma. The present study is aimed to evaluate the psychometric properties of the Chinese version of the PedsQL™ 3.0 Asthma Module.

**Methods:**

The PedsQL™ 3.0 Asthma Module was translated into Chinese following the PedsQL™ Measurement Model Translation Methodology. The Chinese version scale was administered to 204 children with asthma and 337 parents of children with asthma from four Triple A hospitals. The psychometric properties were then evaluated.

**Results:**

The percentage of missing value for each item of the scale ranged from 0.00% to 8.31%. All child self-report subscales and parent proxy-report subscales approached or exceeded the minimum reliability standard of 0.70 for alpha coefficient, except 3 subscales of Young Child (aged 5-7) self-report (alphas ranging from 0.59 to 0.68). Test-retest reliability was satisfactory with intraclass correlation coefficients (ICCs) which exceeded the recommended standard of 0.80 in all subscales. Correlation coefficients between items and their hypothesized subscales were higher than those with other subscales. The PedsQL™ 3.0 Asthma Module distinguished between outpatients and inpatients. Patients with mild asthma reported higher scores than those with moderate/severe asthma in majority of subscales. The intercorrelations among the PedsQL™ 3.0 Asthma Module subscales and the PedsQL™ 4.0 Generic Core Scales were in medium to large effect size. The child self-report scores were consistent with the parent proxy-report scores.

**Conclusions:**

The Chinese version of the PedsQL™ 3.0 Asthma Module has acceptable psychometric properties, except the internal consistency reliability for Young Child (aged 5-7) self-report. Further studies should be focused on testing responsiveness of the Chinese version scale in longitudinal studies, evaluating the reliability and validity of the scale for the patients with severe asthma or teens independently, and assessing HRQOL of children with asthma in other areas.

## Background

Asthma is one of the most common chronic respiratory diseases of childhood in the world [1]. With the aggravation of environmental pollution, the pediatric asthma prevalence rate is increasing significantly in China as well as other countries in the world [1-3]. With the increasing pediatric asthma prevalence rates, more and more asthma-related clinical visits, hospitalizations and mortalities were recorded, resulting in more physical and emotional symptoms, greater activity limitations, and poorer well-being and social functioning for the asthmatic children [1,4,5]. Although the clinical and physiological indicators, such as asthma symptoms and pulmonary function testing, are important, health-related quality of life (HRQOL) can provide a more comprehensive description of the impact of the illness on the life of children with asthma [6]. Measures of HRQOL are also valuable in documenting clinical response to medical treatment and disease-related changes in functioning. A well-developed instrument is of decisive importance for the HRQOL assessment. The instruments of HRQOL in pediatric asthma have been well developed for a few years [7], yet further instrument development was recently emphasized to broaden the age range for both child self-report and parent proxy-report [8]. Moreover, the HRQOL studies were focused on adult populations rather than on children in China. The main reason for that is the lack of suitable instruments, which need to be developed or adapted in Chinese according to the established scientific criteria and attributes [9].

The Pediatric Quality of Life Inventory (PedsQL™) Measurement Model, firstly developed by Varni *et al *in 1999, is a promising instrument to assess HRQOL of children aged 2-18 years [10,11]. With the progressive evaluation and application, a series of scales, including a General Core Scale and several disease-specific modules, have been developed and proved to be reliable and valid [12-14]. The PedsQL™ has been translated into many languages, and been widely used in more than 60 countries [15-17]. In China, the Chinese version of the PedsQL™ 4.0 Generic Core Scale has been developed and psychometrically evaluated [18]. The PedsQL™ 3.0 Asthma Module, one of the disease-specific scales, was designed to measure HRQOL dimensions specifically tailored to pediatric asthma. It has already been adapted to apply in different countries with adequate reliability and validity [19,20]. In order to improve the assessment of the impact of asthma on the pediatric HRQOL in the context with Chinese culture, we decided to generate the Chinese version of the PedsQL™ 3.0 Asthma Module, which could be used in combination with the Chinese version of the PedsQL™4.0 Generic Core Scale with the permission from PedsQL™ copyright owner, James W. Varni.

This study aimed at evaluating the psychometric properties of the Chinese version of the PedsQL™ 3.0 Asthma Module, including the feasibility, internal consistency reliability, test-retest reliability, item-subscale correlations, construct validity and concordance between child self-reports and parent proxy-reports.

## Methods

### Subjects and Settings

Subjects included children with asthma aged 5-18 and parents of children with asthma aged 2-18. The pediatric patients were eligible for the study if they were diagnosed with asthma conforming to the national diagnostic standards of China. The parents were enrolled if they were the parents of children, who were inpatients or outpatients with asthma. Inpatient was defined as a child who was hospitalized for necessary treatment. Outpatient was defined as a child who attended the outpatient department for routine visits. All the subjects were approached with the permission from the doctors. The subjects were excluded if 1) the parents were illiterate or reluctant to participate, or 2) the children were reported to have other chronic diseases or mental disorders.

All subjects were recruited by convenient sampling method from both outpatient and inpatient departments of four Triple A hospitals in Guangzhou, China from December, 2008 to June, 2009. Triple A hospitals are the most-outstanding ones in China, and they provide high-level medical services and implement high medical education and research tasks. This study was approved by the Ethics Committee of School of Public Health, Sun Yat-sen University. Informed consent forms were signed by all subjects.

According to the formats of the PedsQL™ 3.0 Asthma Module, the subjects were divided into four age groups: Toddlers (aged 2-4), Young Children (aged 5-7), Children (aged 8-12) and Teens (aged 13-18). According to the patient recruitment sources, subjects were divided into the inpatient group and the outpatient group. Moreover, according to the asthma severity reported by parents, the subjects were divided into "mild", "moderate" and "severe" asthma groups. The asthma severity was defined by asking parents "How severe do you think the patient's asthma is during the past one month", with three response categories of "mild", "moderate" and "severe".

### Instruments

The Chinese versions of the PedsQL™ 3.0 Asthma Module, 4.0 Generic Core Scale, and Family Information Form were used in this study.

#### PedsQL™ 3.0 Asthma Module

The PedsQL™ 3.0 Asthma Module was developed to measure asthma-specific aspects of HRQOL in children aged 2-18. It is divided into seven forms, including parent proxy-reports for Toddlers (aged 2-4), Young Children (aged 5-7), Children (aged 8-12) and Teens (aged 13-18) and self-reports for Young Children, Children and Teens. Items in all forms are essentially identical, distinguishing only in appropriate language, or first- or third-person tense. This 28-item instrument consists of 4 subscales: Asthma Symptoms (11 items), Treatment Problems (11 items), Worry (3 items), and Communication (3 items). The instructions ask how much of a problem each item has been during the past one month. Responses are rated on a 5-point Likert scale across child self-report for Children and Teens as well as parent proxy-report (0 = never a problem, 1 = almost never a problem, 2 = sometimes a problem, 3 = often a problem, 4 = almost always a problem). A 3-point scale (0 = Not at all, 2 = Sometimes, 4 = A lot) is utilized specifically for the child self-report for Young Children. Items are reversed scored and linearly transformed to a 0-100 scale (0 = 100, 1 = 75, 2 = 50, 3 = 25, 4 = 0), so that higher scores indicate better HRQOL. Subscale scores are computed as the sum of the items divided by the number of items answered. If more than 50% items in the scale are missing, the subscale scores would not be computed [21].

#### PedsQL™4.0 Generic Core Scale

The PedsQL™4.0 Generic Core Scale is an instrument with 23 items grouped into four subscales: Physical Functioning (8 items), Emotional Functioning (5 items), Social Functioning (5 items) and School Functioning (5 items). The formats, instructions, Likert scales, and scoring methods are the same as those of the PedsQL™ 3.0 Asthma Module. In addition to the four subscale scores, three types of summary scores can be obtained in the PedsQL™4.0 Generic Core Scale: 1) Physical Health Summary Score equals Physical Functioning subscale score; 2) Psychosocial Health Summary Score is calculated as the sum of the 15 items of Emotional, Social, and School Functioning subscales divided by the number of items answered; 3) Total Score is calculated as the sum of all 23 items divided by the number of items answered.

#### PedsQL™ Family Information Form

The PedsQL™ Family Information Form, completed only by parents, contains general socio-demographic information including the child's date of birth, gender, disease history, disease severity and the parent's marital status, education, occupation, family income, and payment method for the child's medical care.

#### Cross-culture adaptation

The PedsQL™ Measurement Model Translation Methodology was strictly followed in the linguistic translation process of the PedsQL™ 3.0 Asthma Module in this study [22]. It was summarized as the procedure of "Forward Translation - Backward Translation - Preliminary Test - Field Test".

The forward translation was performed by a pediatrician and a medical English teacher independently, both of whom were fluent in English. A multidisciplinary team including a pediatrician, a nurse, a health services researcher and the project manager who was also a statistician, then reviewed the two drafts. They compared the first two drafts, and made decisions on which translation was more equivalent to the original meaning and suitable for Chinese. A single reconciled Chinese version was developed after discussion.

The backward translation was performed by a bilingual pediatrician who was working in the United States. He was a native Chinese speaker and also was fluent in English, and was not aware of the instrument before. The backward translated version was compared with the original one by the multidisciplinary team. Any inaccuracy or disaccord would be rectified to assure semantic and conceptual equivalence. Then, the second Chinese version was yielded.

Cognitive debriefing was conducted in 20 pediatric patients with asthma and their parents. It was to confirm that the final Chinese version was understandable and acceptable. This preliminary test was performed by face-to-face interviews in order to obtain comments and suggestions on the Chinese scale from interviewees. After some necessary revisions, the Chinese version scale was finalized and approved for field-testing in the current study.

All stages' reports were sent to and accepted by Mapi research Institute in Lyon, France, on behalf of Dr. James W. Varni, the copyright owner of the PedsQL™.

### Data collection

Five undergraduate students majoring in Preventive Medicine and two nurses were trained as interviewers by the project manager before the investigation. The parents and their children completed the questionnaire independently during the pediatric patients' hospitalization or outpatient department visit. All the parents were asked to fill out the PedsQL™ 3.0 Asthma Module, the 4.0 Generic Core Scale, and the Family Information Form by self-administration. The children were required to complete the PedsQL™ 3.0 Asthma Module and the 4.0 Generic Core Scale by self-administration except the Young Children by interview-administration. The interviewers were available to assist the completion of the questionnaires if the parents/children had questions on semantic or conceptual understanding. They were also responsible for collecting and checking the questionnaires to ensure that there were no missing data or logical mistakes. With the purpose of evaluating the test-retest reliability of the scale, the PedsQL™ 3.0 Asthma Module was administered repeatedly to 50 compliable hospitalized patients who had stable asthma symptoms one week after the first interview.

### Data Analysis

Data were analyzed with SPSS 13.0 for windows. Descriptive analysis was used for reporting the socio-demographic characteristics of the parents and children. Continuous variables were presented as mean and standard deviation (). Categorical variables were shown as observed frequencies and proportions. The presence of floor and ceiling effects (>25% of the respondents have the minimum and/or maximum score) was evaluated for the four subscale scores [23].

The response rate of the Asthma Module was also calculated in this study. It was defined as the number of subjects in the analysis divided by the number of subjects approached for the study.

Feasibility was determined by the average completion time and percentage of missing value for each item. The average completion time was defined as the mean of completion time of the Asthma Module. The percentage of missing value for each item was defined as the number of subjects who did not fill out the item divided by the number of eligible subjects who were supposed to complete the item.

Subscale internal consistency reliability was determined using Cronbach's alpha coefficient. Subscales with alpha ≥0.70 were recommended for comparing patient groups, while a reliability criterion of 0.90 is recommended for analyzing individual patient [24]. Intraclass correlation coefficient (ICC) was used to evaluate the test-retest reliability for the subscales. Values greater than 0.80 indicated high test-retest reliability [25].

Multitrait scaling analysis (using Pearson correlation analysis) was conducted to determine the item-subscale correlations. Good scaling success was indicated if the correlations between item and its hypothesized subscale were stronger than those with other subscales.

Construct validity for the PedsQL™ 3.0 Asthma Module was evaluated by means of the known-groups method, which compares subscale scores across groups that are known to differ in the health conditions being investigated [14]. In this study, the independent sample *t *test was used to compare: 1) children who were inpatients versus those who were outpatients, 2) children with mild asthma versus those with moderate/severe asthma. It was hypothesized that outpatients would have higher HRQOL than inpatients, based on previous findings on other PedsQL™ scales and the current literatures regarding the correlations among the hospitalization, the adverse outcome of asthma and the conceptualization of HRQOL, which was a marker of disease severity [14,26-29]. It was also hypothesized that the children with mild asthma would have higher subscale scores than children with moderate/severe asthma, based on the previous findings on the association between the adverse asthma outcome and the patient's HRQOL [30].

Construct validity for the PedsQL™ 3.0 Asthma Module was further evaluated through analyses of the intercorrelations among the PedsQL™ 3.0 Asthma Module subscale scores and the PedsQL™ 4.0 Generic Core Scale Total Score. It had been reported that computing the intercorrelations among scales provides initial information on the construct validity of an instrument [14]. Correlation effect sizes were designed as small (0.10-0.29), medium (0.30-0.49), and large (≥0.50) [31]. Intercorrelations were expected to demonstrate medium to large effect size [25]. On the grounds that disease-specific symptoms could be used as causal indicators of generic HRQOL [25], it was hypothesized that higher Asthma Symptom subscale score (fewer symptoms) would be correlated with higher Generic Core Scale Total Score (better overall HRQOL). Based on the previous findings on the association between disease-specific side effects or barriers to treatment adherence and patient's generic HRQOL [32], it was hypothesized that higher Treatment Problems subscale score (fewer treatment side effects or barriers to adherence) would be correlated with higher Generic Core Scale Total Score (better overall HRQOL). It was further hypothesized that higher Worry and Communication subscale scores (less worry and better communication respectively) would be correlated with higher Generic Core Scale Total Score (better overall HRQOL) based on the previous findings on other PedsQL™ disease-specific module [33].

The concordance between self-reports and proxy-reports was evaluated by ICC and paired sample *t *tests. ICCs were designated as poor to fair agreement (≤0.40), moderate agreement (0.41 to 0.60), good agreement (0.61 to 0.80), and excellent agreement (>0.80) [34]. Additionally, parent-child intercorrelations were computed to examine cross-informant variance. Correlation effect sizes are designed as small (0.10-0.29), medium (0.30-0.49), and large (≥0.50) [31]. Parent-child concordance for the same subscale score was expected to demonstrate medium to large effect size, but not so large that child and parent reports would be redundant, based on previous findings of PedsQL™ research studies [33,35].

## Results

### Subjects

Participants were children with asthma (*n *= 204) and parents of children with asthma (*n *= 337) approached for the study, with 337 families collected overall. For 204 children aged 5-18, both child self-report and parent proxy-report were available, while only parent proxy-report were available for 133 children aged 2-4.

The average age of the pediatric patients was 6.36 years (*SD *= 2.96) with a range of 2.00 to 14.30 years. A total of 232 of the patients were boys, 104 were girls and 1 child's gender was not reported. Of the pediatric patients, 39.47% were Toddlers, 30.86% were Young Children, 27.00% were Children, and 2.67% were Teens. In order to guarantee the power of test, the groups of Children and Teens were combined into one group for subsequent analyses because of the small sample size of Teens (*n *= 9). In this study, the majority of the patients (67.36%) suffered from mild asthma, while others suffered from moderate and severe asthma (29.67% and 2.97% respectively). Since the sample size of patients with severe asthma was small (*n *= 10), we combined the moderate asthma group with severe asthma group to ensure the power of test in the analyses. All the pediatric patients (included 50 inpatients and 287 outpatients) had an average history of asthma of 3.06 years (*SD *= 2.33). A total of 337 parents participated in the study. Detailed sample characteristics are presented in Table 1.

**Table 1:** Demographic Characteristics of the Samples

Demographic Characteristics	*N*	%
***Characteristics of Parents***		
**Relationship to Patient**		
Mother	247	73.29
Father	67	19.88
Others	23	6.83
***Characteristics of Children***		
**Ages (years)**		
2~4*	133	39.47
5~7	104	30.86
8~12	91	27.00
13~18	9	2.67
**Gender**		
Male	232	68.84
Female	104	30.86
Not reported	1	0.30
**Groups**		
Inpatient	50	14.84
Outpatient	287	85.16
**Disease Severity**		
Mild	227	67.36
Moderate	100	29.67
Severe	10	2.97

* The information of Toddlers was offered by their parents.

### Descriptive Analysis

Table 2 displays means, standard deviations, floor effects and ceiling effects on each subscale score of the PedsQL™ 3.0 Asthma Module. The Asthma Module show ceiling effects in all subscale scores except Asthma Symptoms subscale score. Additionally, patients with mild disease had greater ceiling effects than patients with moderate/severe disease. However, there was no floor effect in all subscale scores. The means and standard deviations for inpatients, outpatients and patients with different disease severity are presented in Table 3.

**Table 2:** Subscales descriptives for the PedsQL™ 3.0 Asthma Module for the self-report and proxy-report

Subscale	Number of items	*N*	Mean	*SD*	% Floor*			% Ceiling*		
					
					Total sample	Mild	Moderate /Severe	Total sample	Mild	Moderate /Severe
**Self-Report**										
Asthma Symptoms	11	204	81.23	12.09	0.0	0.0	0.0	3.4	5.0	0.0
Treatment Problems	11	204	91.46	9.47	0.0	0.0	0.0	30.4	36.2	17.5
Worry	3	201	84.89	18.45	0.0	0.0	0.0	46.6	49.6	39.7
Communication	3	204	88.97	19.38	1.0	0.0	1.4	64.2	66.7	58.7
**Proxy-Report**										
Asthma Symptoms	11	337	79.72	14.29	0.0	0.0	0.0	4.5	6.6	0.0
Treatment Problems	11	337	89.78	12.19	0.0	0.0	0.0	36.2	43.2	21.8
Worry	3	311	87.45	21.07	1.0	1.0	1.0	64.3	73.3	46.7
Communication	3	318	86.61	21.57	1.2	0.9	1.9	61.3	67.0	50.0

*% Floor/%Ceiling = the percentage of scores at the extremes of the scaling range

*SD *= standard deviation

**Table 3:** Construct Validity of the Chinese Version Scale Assessed by the Known-groups Method (Mean(SD))

Subscales	Sample groups					Disease severity				
	
	N *	inpatient	outpatient	*t *†	*P *‡	*N *§	mild	moderate /severe	*t *†	*P *‡
**self-report**										
Asthma Symptoms	28/176	69.16 (15.49)	83.15 (10.27)	6.19	<0.001	141/63	84.56 (9.58)	73.77 (13.77)	6.45	<0.001
Treatment Problems	28/176	86.68 (11.63)	92.22 (8.88)	2.93	0.004	141/63	92.74 (8.65)	88.58 (10.61)	2.96	0.003
Worry	28/176	73.21 (21.91)	86.74 (17.19)	3.72	<0.001	141/63	86.29 (18.29)	81.75 (18.57)	1.63	0.104
Communication	28/173	81.55 (28.63)	90.17 (17.26)	2.21	0.029	138/63	89.73 (19.03)	87.30 (20.18)	0.82	0.410
**proxy-report**										
Asthma Symptoms	50/287	68.41 (13.66)	81.69 (13.47)	6.42	<0.001	227/110	86.33 (9.99)	66.07 (11.98)	16.33	<0.001
Treatment Problems	50/287	81.05 (13.24)	91.30 (11.35)	5.75	<0.001	227/110	92.34 (10.50)	84.50 (13.70)	5.80	<0.001
Worry	50/261	73.83 (21.89)	90.05 (19.92)	5.19	<0.001	206/105	91.02 (18.90)	80.44 (23.34)	4.30	<0.001
Communication	40/269	74.66 (25.51)	88.79 (20.07)	4.33	<0.001	212/106	89.07 (20.29)	81.68 (23.24)	2.91	0.004

* inpatients/outpatients

§ mild/moderate/severe

† independent sample *t *test

‡ *P *values for the *t *test

### Response Rate and Feasibility

The response rate for the children and the parents were 97.61% and 98.83% respectively. There were 209 children with asthma and 341 parents of children with asthma participating in the study. A total of 204 children completed the questionnaire, 4 children refused to participate and 1 child answered less than 50% of the items. A total of 337 parents completed the questionnaire, 4 parents refused to participate since they were in a rush or unwilling to do it. The average completion time of the PedsQL™ 3.0 Asthma Module was about 5 minutes. The percentage of missing value for each item of the scale ranged from 0.00% to 8.31% (Table 4).

**Table 4:** Item-subscale Correlations of the PedsQL™ 3.0 Asthma Module^§^

Subscales & Items	Parent proxy-report					Child self-report				
	
	A	TX	W	C	missing (n/%)	A	TX	W	C	missing (n/%)
**Asthma Symptoms (A)**										
1. pain or tightness in his or her chest	0.68	0.34	0.35	0.18	0/0.00	0.50	0.33	0.28	0.08*	0/0.00
2.feeling wheezy	0.73	0.37	0.32	0.22	0/0.00	0.65	0.29	0.32	0.10*	0/0.00
3. having asthma attacks	0.75	0.39	0.34	0.22	0/0.00	0.67	0.38	0.17	0.01*	0/0.00
4.getting scared while having asthma attacks	0.65	0.41	0.34	0.25	0/0.00	0.43	0.29	0.17	0.04*	0/0.00
5.getting out of breath	0.77	0.46	0.39	0.28	0/0.00	0.62	0.32	0.40	0.18	0/0.00
6.coughing	0.60	0.31	0.16	0.19	0/0.00	0.44	0.24	0.14	0.03*	0/0.00
7.taking a deep breath	0.70	0.44	0.36	0.32	0/0.00	0.55	0.38	0.21	0.09*	0/0.00
8. having a stuff or runny nose	0.52	0.25	0.14	0.20	0/0.00	0.47	0.33	0.07*	0.25	0/0.00
9.waking up at night with trouble breathing	0.73	0.46	0.32	0.36	0/0.00	0.47	0.31	0.22	0.0.9*	0/0.00
10.playing with pets	0.53	0.48	0.32	0.35	1/0.30	0.37	0.17	0.16	0.15	1/0.49
11. playing outside	0.61	0.53	0.39	0.30	0/0.00	0.48	0.19	0.25	0.11*	0/0.00
**Treatment Problems (TX)**										
1.medicines making him or her feel sick	0.42	0.62	0.37	0.32	0/0.00	0.32	0.56	0.27	0.25	0/0.00
2.trouble sleeping because of medicines	0.43	0.60	0.29	0.34	0/0.00	0.20	0.33	0.12*	0.15	0/0.00
3. trouble using his or her inhaler	0.35	0.56	0.26	0.26	10/2.97	0.31	0.52	0.11*	0.23	7/3.43
4.disliking carrying his or her inhaler	0.33	0.57	0.27	0.36	9/2.67	0.21	0.47	0.06*	0.29	7/3.43
5.being responsible for his or her medicines	0.39	0.65	0.37	0.31	0/0.00	0.32	0.58	0.22	0.22	0/0.00
6.controlling his or her asthma	0.64	0.67	0.33	0.26	0/0.00	0.53	0.60	0.36	0.23	0/0.00
7.refusing to take medicines	0.29	0.66	0.33	0.28	0/0.00	0.23	0.51	0.12*	0.10*	0/0.00
8.forgetting to take medicines	0.31	0.61	0.37	0.36	1/0.30	0.21	0.51	0.24	0.13*	0/0.00
9.getting anxious when he or she has to have medical treatments	0.40	0.73	0.50	0.37	0/0.00	0.29	0.49	0.30	0.27	0/0.00
10.getting anxious about going to the doctor	0.40	0.68	0.40	0.34	0/0.00	0.26	0.43	0.14	0.07*	0/0.00
11.getting anxious about going to the hospital	0.38	0.68	0.49	0.36	0/0.00	0.23	0.43	0.22	0.08*	0/0.00
**Worry (W)**										
1.worrying about side effects from medical treatments	0.39	0.51	0.91	0.28	28/8.31 ‡	0.34	0.31	0.69	0.15	0/0.00
2.worrying about whether or not medical treatments are working	0.42	0.53	0.93	0.38	28/8.31 ‡	0.28	0.35	0.77	0.25	0/0.00
3.worrying about his or her asthma	0.42	0.52	0.92	0.31	22/6.53 ‡	0.35	0.28	0.83	0.08*	0/0.00
**Communication (C)**										
1.telling the doctors and nurses how he or she feels	0.35	0.47	0.32	0.91	16/4.75 ‡	0.16	0.36	0.14	0.82	2/0.98
2.asking the doctors or nurses questions	0.34	0.46	0.32	0.96	23/6.82 ‡	0.14*	0.27	0.17	0.87	7/3.43
3.explaining his or her illness to other people	0.37	0.49	0.36	0.93	20/5.93 ‡	0.22	0.35	0.19	0.90	2/0.98

§ Values denote Pearson correlation coefficients. The *P *value of all coefficients above is less than 0.05, except that noted by "*".

Underlined values represent correlations between items and their hypothesized subscales.

‡ For the Worry and Communication subscales (parent proxy-report), the higher percentage of missing values is primarily from the Toddler version.

### Reliability

Cronbach's alpha coefficients for the PedsQL™ 3.0 Asthma Module across all ages are presented in Table 5. For the total sample, all coefficients were higher than 0.70 in all subscales except the Treatment Problems and Worry subscales (*α *= 0.69 and 0.65 respectively) in the child self-report. The subscales of child self-report and parent proxy-report across all ages approached or exceeded the minimum reliability standard 0.70 except 3 subscales of Young Child self-report. The ICCs being used to examine the test-retest reliability were all higher than 0.80 in all subscales (Table 5).

**Table 5:** Reliability for PedsQL™ 3.0 Asthma Module for self and proxy-reports

Subscale	Age group (α *)				Age group(ICC^§^)			
	
	Toddlers (2-4)	Young Children (5-7)	Children & Teens (8-18)	Total Sample	Toddlers (2-4)	Young Children (5-7)	Children & Teens (8-18)	Total Sample
**Self-Report**								
Asthma Symptoms	N/A	0.65	0.78	0.72	N/A	0.95	0.88	0.92
Treatment Problems	N/A	0.59	0.77	0.69	N/A	0.79	0.93	0.89
Worry	N/A	0.68	0.62	0.65	N/A	0.80	0.95	0.90
Communication	N/A	0.86	0.77	0.82	N/A	0.97	0.96	0.97
**Proxy-Report**								
Asthma Symptoms	0.88	0.86	0.87	0.87	0.94	0.91	0.94	0.93
Treatment Problems	0.81	0.86	0.84	0.85	0.91	0.96	0.91	0.92
Worry	0.91	0.91	0.91	0.91	0.88	0.96	0.95	0.92
Communication	0.94	0.89	0.92	0.93	0.99	0.99	0.96	0.99

* *α *= Cronbach's alpha coefficients

§ ICC = Intraclass correlation coefficient

N/A = not applicable

### Item-subscale correlations

Pearson correlation coefficients between items and subscale scores are presented in Table 4. The result showed that items had moderate to strong correlations with their hypothesized subscales, which were higher than those with other subscales (*P *< 0.05).

### Construct validity

Construct validity of the PedsQL™ 3.0 Asthma Module assessed by the known-groups method is presented in Table 3. For every comparison for subscale scores, there was a statistically significant difference between inpatients and outpatients (*P *< 0.05). Furthermore, the children with mild asthma reported significantly higher subscale scores than the children with moderate/severe asthma in most of the subscales (*P *< 0.05).

The result of the intercorrelations between the PedsQL™ 3.0 Asthma Module subscale scores and the PedsQL™ 4.0 Generic Core Scale Total Score is shown in Table 6. The correlations were in medium to large effect size, with largest intercorrelations between the PedsQL™ 3.0 Asthma Module Asthma Symptoms subscale score and the PedsQL™ 4.0 Generic Core Scales Total Score for child and parent report (*r *= 0.64 and *r *= 0.65, respectively).

**Table 6:** Pearson Correlations Coefficients Among PedsQL™ Subscales*

Subscales	Tot	PH	PSY	EM	SOC	SCH	A	TX	W	C
Total score (Tot)	0.44	0.82	0.94	0.76	0.71	0.72	**0.64**	**0.59**	**0.45**	**0.44**
Physical Health (PH)	0.85	0.45	0.58	0.46	0.45	0.45	0.57	0.47	0.37	0.34
Psychosocial Health (PSY)	0.96	0.67	0.40	0.81	0.76	0.76	0.57	0.57	0.43	0.43
Emotional Functioning (EM)	0.79	0.52	0.84	0.25	0.48	0.39	0.51	0.54	0.39	0.34
Social Functioning (SOC)	0.81	0.62	0.81	0.55	0.36	0.35	0.36	0.39	0.31	0.35
School Functioning (SCH)	0.74	0.49	0.78	0.43	0.50	0.49	0.45	0.39	0.29	0.31
Asthma Symptoms (A)	**0.65**	0.60	0.60	0.51	0.44	0.51	0.56	0.58	0.42	0.20
Treatment Problems (TX)	**0.60**	0.46	0.60	0.56	0.49	0.42	0.59	0.30	0.40	0.38
Worry (W)	**0.41**	0.34	0.40	0.34	0.35	0.29	0.46	0.56	0.32	0.20
Communication (C)	**0.37**	0.31	0.36	0.31	0.37	0.26	0.38	0.51	0.35	0.27

*Correlations for patient self-report are shown above the diagonal.

Underlined values represent correlations between patient self-report and parent proxy-report.

Correlations for parent proxy-report are shown below the diagonal.

Correlations among Generic Core Scale Total Score with the Asthma Module subscale scores are in boldface.

Effect size are designated as small (0.10-0.29), medium (0.30-0.49), and large (≥0.50)

### Self-report/Proxy-report concordance

The parent/child concordance intercorrelations matrix is shown in Table 6. Most intercorrelations of subscales between child self-report and parent proxy-report were in medium to large effect size range.

The result of paired sample *t *tests for the total sample showed that there was no significant difference between the subscale scores of self-reports and those of proxy-reports. The values of ICCs were all greater than 0.77 (shown in Table 7).

**Table 7:** The Concordance between Self-report and Proxy-report of the PedsQL™ 3.0 Asthma Module

Subscales	Total sample			Young Children (5-7)			Children &Teens (8-18)		
	
	t*	*P*^§ ^	ICC	t*	*P *	ICC	t*	*P *	ICC
Asthma Symptoms	-1.35	0.177	0.87	-2.31	0.023	0.84	0.57	0.570	0.89
Treatment Problems	-0.97	0.334	0.80	-1.69	0.094	0.80	0.18	0.856	0.81
Worry	1.19	0.237	0.77	0.11	0.909	0.78	1.51	0.135	0.77
Communication	-0.39	0.697	0.81	-1.16	0.250	0.85	0.58	0.562	0.75

* paired sample *t *test

§ *P *values for the *t *tests

ICC = Intraclass correlation coefficient

## Discussion

The PedsQL™3.0 Asthma Module, one of the PedsQL™disease-specific modules, is designed to measure the asthma specific HRQOL of pediatric patients. With satisfactory psychometric properties, both the child self-report and the parent proxy-report, being available, brief and applicable for children with a broad age range, make the PedsQL™ a good HRQOL measurement. Additionally, while there are a number of asthma disease-specific instruments available [8], there are potential benefits of integrating generic and disease-specific approaches in measuring HRQOL [10]. The development of the Chinese version of the PedsQL™3.0 Asthma Module will not only meet an urgent demand in the HRQOL assessment in children with asthma in China, but also make it possible to compare their HRQOL across countries.

This study presents the measurement properties for the Chinese version of the PedsQL™3.0 Asthma Module. To our knowledge, this is also the first time that the PedsQL™3.0 Asthma Module was tested on children with asthma in China. The analyses support the feasibility, reliability and validity of the PedsQL™3.0 Asthma Module as a child self-report and parent proxy-report HRQOL measurement instrument for pediatric asthma.

The Chinese version of the PedsQL™3.0 Asthma Module, developed strictly following the PedsQL™ Measurement Model Translation Methodology, was feasible and practical. In particular, short completing time made this instrument particularly applicable to the fast-pace setting of an outpatient clinic. However, it is found that there were several items of Worry and Communication subscales in the parent proxy-report, i.e. "worrying about side effects from medical treatments", had a high missing rate. We also found that the higher percentage of missing values was primarily from the Toddler version. The reason may be that, parents regarded their children as too young to understand the medical treatment effect and side effect or communicate with doctors and nurses. This finding was consistent with those of the prior studies with other PedsQL™disease-specific modules [33]. This indicated that some modifications for the items of Worry and Communication subscales in Toddler version scale were necessary. In addition, ceiling effect was found in the study, but no floor effect was observed in most of the PedsQL™3.0 Asthma Module subscales. Considering that the majority of patients suffering from mild asthma (about 70%) might account for the high ceiling effect, exploratory analyses were conducted for patients with different disease severity. We found that patients with mild disease had greater ceiling effects than patients with moderate to severe disease. This suggested that the instrument could show the HRQOL changes in patients who were experiencing adverse impacts from their asthma even though it might not be sensitive to perceive HRQOL improvement in patients who were relatively doing well.

Internal consistency reliability was evaluated using Cronbach's alpha coefficients. Consistent with the coefficients reported by Varni *et al *[14], all the Cronbach's alpha coefficients (except Treatment Problems and Worry subscales in the child self-report) exceeded the recommended minimum alpha coefficient standard of 0.7 for group comparisons, indicating acceptable reliability of the Chinese version scale. Considering that scores may be age-dependent, exploratory analyses were conducted for different age groups. The internal consistency reliabilities of the PedsQL™3.0 Asthma Module subscales across all ages generally approached or exceeded the recommend standard of 0.7 except for the Young Child self-report. The coefficients did not achieve the standard value probably resulting from the small sample size (*n *<100). On the other hand, the bias due to different modes of administration could not be excluded. The Young Children completed the scale by face-to-face interviews which might bias the answers due to misunderstanding, even though all the investigators were trained in advance. It's suggested that the subscales that did not achieve the standard should be used only for descriptive or exploratory analyses unless updated findings are obtained in further testing [14].

Test-retest reliability was examined by computing ICC. For the total sample and the patients of different age groups, the ICCs were close to or higher than the recommend standard of 0.8, indicating a good test-retest reliability of the scale. This finding demonstrated that the Chinese version of the PedsQL™3.0 Asthma Module was stable over time.

In order to evaluate the item-subscale correlations, Pearson correlations between items and subscale scores were analyzed. The results of the correlations between items and their hypothesized subscales being high but those between items and other subscales being weak indicated good scaling success for child self-reports and parent proxy-reports.

Construct validity of the Chinese version of the PedsQL™3.0 Asthma Module was assessed by means of known-groups method. The hypothesis was supported that the outpatients had higher HRQOL than inpatients. Another hypothesis that the children with mild asthma would manifest higher HRQOL than the children with moderate/severe asthma was also verified in all subscales except child self-report Worry and Communication. The subscales with unqualified discriminate ability, which was probably yielded by the relatively small sample size of the children with moderate/severe asthma (*n *= 63), were recommended for further testing. In addition, the children's asthma severity determined based on parent-reported information might be less reliable than that categorized by the clinical and physiological indicators, such as asthma symptoms and pulmonary function testing [36]. This might partly accounted for the unqualified discriminate ability of the Asthma Module.

Construct validity was further tested by analyzing the intercorrelations among the Chinese version of the PedsQL™ 3.0 Asthma Module subscale scores and the validated Chinese version of the PedsQL™ 4.0 Generic Core Scale Total Score. Similar to the findings in a previous study [14], the intercorrelations between the PedsQL™ 4.0 Generic Core Scale Total Score and PedsQL™ 3.0 Asthma Module subscale scores were consistent with the conceptualization of disease-specific symptoms as causal indicators of HRQOL.

Based on the results of paired sample *t *tests and ICCs, it was believed that good concordance existed in child self-reports and parent proxy-reports. Moreover, the cross-informant variance was observed in the parent-child intercorrelations matrix. That supports the need to measure the perspectives of child and parent informants in evaluating HRQOL in pediatric asthma. This finding was consistent with that of a previous study [14]. The validated parent proxy-report can be used to estimate pediatric HRQOL when the child is unable or unwilling to complete the HRQOL measurement, or treated as proxy information when young child self-report scale reliabilities do not achieve the standard [14].

Several limitations in this study should be considered. Firstly, the responsiveness of the Chinese version of the PedsQL™3.0 Asthma Module was not evaluated in the study. Responsiveness which is used to detect the HRQOL changes while a patient's health status changes over time can be regarded as providing additional evidence of validity of an instrument [25]. Further longitudinal studies were advised to assess the responsiveness. Secondly, the sample size of the severe asthma group and the Teens group was very small. In order to guarantee the power of test, we combined the moderate asthma group and the severe asthma group, as well as the Children group and Teens group, into one group for subsequent analyses. Thus, the reliability and validity of the scale for the patients with severe asthma or teens need to be further confirmed independently. Thirdly, all the subjects in this study were recruited in a large city (Guangzhou) in China. Thus, whether the Chinese version of the PedsQL™ 3.0 Asthma Module can be generalized to other regions remains a question. Further studies conducted in other areas are suggested.

## Conclusions

This study is important as being the first time to introduce the PedsQL™ 3.0 Asthma Module to China and evaluate the psychometric properties of the Chinese version scale based on the Chinese pediatric asthma patients. The data collected by our study provide reasonable evidence to show that the Chinese version of the PedsQL™ 3.0 Asthma Module has acceptable psychometric properties except the internal consistency reliability for Young Child self-report. Further studies should focus on testing responsiveness of the Chinese version scale in longitudinal studies, evaluating the reliability and validity of the scale for the patients with severe asthma or teens independently, and assessing HRQOL of children with asthma in other areas.

## List of abbreviations

PedsQL™: Pediatric Quality of Life Inventory™; HRQOL: Health-Related Quality of Life; ICC: Intraclass correlation coefficient.

## Competing interests

The authors declare that they have no competing interests.

## Authors' contributions

LF conceptualized and designed the study, acquired, analyzed and interpreted the data, and drafted the manuscript. YZ conceptualized and designed the study, acquired, analyzed and interpreted the data, and revised the manuscript. RC conceptualized and designed the study, acquired data, and revised the manuscript. YH conceptualized and designed the study, supervised the data analysis and revised the manuscript. All authors read and approved the final manuscript.
